# Exosomes in Myasthenia Gravis—Review

**DOI:** 10.3390/cells15080679

**Published:** 2026-04-13

**Authors:** Krystian Ejdys, Marcin P. Mycko

**Affiliations:** Department of Neurology and Neurosurgery, Laboratory of Neuroimmunology, University of Warmia and Mazury in Olsztyn, 10-082 Olsztyn, Poland; marcin.mycko@uwm.edu.pl

**Keywords:** myasthenia gravis, exosomes, extracellular vesicles, miRNA, biomarkers

## Abstract

**Highlights:**

**What are the main findings?**

- Circulating blood exosomes are associated with the immune process in myasthenia gravis.
- They may represent a minimally invasive source of biomarkers for the diagnosis of this condition.

**What are the implications of the main findings?**

- Exosomes in myasthenia gravis (MG) may provide additional insight into disease mechanisms and may support future studies on disease monitoring.
- The unique properties of exosomes suggest potential relevance for future research into the modulation of autoimmune responses in MG.

**Abstract:**

Myasthenia gravis (MG) is a rare autoimmune disorder characterized by muscle weakness and fatigue, caused by autoantibodies produced by B-cells that target proteins in the postsynaptic membrane of the neuromuscular junction. Clinical manifestations are heterogeneous and may include diplopia, ptosis, dysarthria, dysphagia, and limb muscle weakness, with severity ranging from mild symptoms to life-threatening myasthenic crisis. Despite advances in diagnostic approaches and the availability of immunomodulatory and biological therapies, there remains a need for an improved understanding of the disease mechanisms and biomarker development in MG. Blood-derived exosomes are small extracellular vesicles that carry proteins, lipids, nucleic acids, and glycoconjugates, and are involved in intercellular communication and the transfer of biological material between cells. Circulating exosomes may reflect aspects of cellular and immune status and have been proposed as a minimally invasive source of biomarkers in various diseases. In this review, we summarize current evidence on the potential role of exosomes in MG, with a focus on their involvement in disease-associated processes and their possible utility as biomarkers, as well as directions for future research.

## 1. Introduction

Myasthenia gravis (MG) is a rare, autoimmunological disease characterized by muscle weakness and fatigue, caused by B lymphocytes and associated with antibodies directed against the acetylcholine receptor, muscle-specific kinase (MuSK), lipoprotein-related protein 4 (LRP4), or agrin in the postsynaptic membrane of the neuromuscular junction [1]. Some patients are suffering from MG despite the lack of evidence for the presence of antibodies to any of these autoantigens (triple seronegative MG; SN) [1]. In the United States of America (2021), the overall incidence of MG was 3.2 per 100,000, with similar estimates for men and women (3.2 vs. 3.1 per 100,000, respectively). The overall prevalence was estimated at 37.0 per 100,000, with comparable gender estimates of 37.3 and 36.7 per 100,000 for men and women, respectively [2]. Simultaneously, studies in Europe reported incidence rates between 0.63 and 2.9 per 100,000 person-years and prevalence rates between 11.17 and 36.1 per 100,000 persons [3]. Thus MG represents a major neurological problem that is still not fully satisfactorily managed.

Beyond classical immunological mechanisms, increasing evidence suggests that intercellular communication networks may also contribute to MG pathophysiology. In this review, the term “extracellular vesicles (EVs)” is used as a general descriptor, while the terminology applied in individual studies is retained to reflect the original authors’ definitions.

## 2. Methods

This narrative review was conducted to provide an up-to-date overview of the role of extracellular vesicles, with a particular focus on exosomes, in myasthenia gravis. A structured literature search was primarily performed using the PubMed database to identify relevant articles published up to January 2026. No lower date limit was applied in order to capture both early and recent developments in the field. The search strategy included combinations of the following terms: “myasthenia gravis”, “extracellular vesicles”, “exosomes”, and “microRNA” (miRNA). To ensure comprehensive coverage, the reference lists of selected articles were manually screened to identify additional relevant studies. The selection of publications was based on their relevance to the pathophysiology of myasthenia gravis, circulating exosomal biomarkers, and experimental therapeutic approaches. Both original research articles and selected review papers were considered, with priority given to studies involving human subjects as well as mechanistic insights from animal models. Given the narrative nature of this review, no formal systematic review protocols or predefined inclusion/exclusion criteria were applied. However, efforts were made to include representative and up-to-date studies to minimize selection bias.

## 3. Myasthenia Gravis Clinical Symptoms and Therapeutic Management

### 3.1. Clinical Symptoms

Taking into account the age of prevalence of MG we classify patients into subgroups: early-onset MG (EOMG), which usually presents in the second to fourth decade of life, is associated with thymic hyperplasia and is more frequent in women; and late onset MG (LOMG), which typically starts around 70 years of age, is not associated with a defined thymus pathology and is slightly more common in men [4]. Symptoms of the disease can be varied and include: muscle weakness, double vision, drooping eyelids, difficulty speaking, dysarthria, difficulty swallowing (dysphagia) or muscle weakness in the limbs. The severity of these symptoms may range from mild to very severe conditions, which can be life-threatening (MG crisis). Myasthenic crisis (MC) occurs in approximately 15–20% of patients with MG, with a mortality of MC in US hospitals of approximately 4.5%; the data on mortality worldwide range between 2 and 16% [5]. The simplest division of clinical types in myasthenia gravis is into ocular and generalized MG. Ocular myasthenia gravis (OMG) represents the most common initial manifestation of myasthenia gravis (MG) and is characterized by ptosis and diplopia. Although the majority of patients presenting with ocular symptoms at disease onset subsequently progress to generalized MG, most commonly within the first two years, OMG is defined as MG restricted exclusively to ocular involvement for a minimum duration of two years. Generalized myasthenia gravis is characterized by widespread skeletal muscle involvement extending beyond the ocular muscles. Proximal muscles are more commonly and severely affected than distal muscles, leading to functional impairments such as difficulty lifting objects, climbing stairs, or raising the arms. The involvement of bulbar muscles results in symptoms including dysphagia and dysarthria [6].

The commonly used classification of patients’ symptom severity with MG is the MGFA classification [7]. The classification consists of five classes, in which class I is ocular muscle weakness only; class II—ocular and mild systemic weakness; class III—ocular and moderate systemic weakness; class IV—ocular and severe systemic weakness; and class V—myasthenic crisis with respiratory failure requiring intubation [8,9]. Other important scales to assess MG patients are: the quantitative myasthenia gravis score (QMG)—a 13-item scale measuring muscle strength and endurance, with scores ranging from 0 to 39, with higher scores indicating greater disease activity [10]; the myasthenia gravis activities of daily living profile (MG-ADL)—an eight-item patient-reported scale designed to assess MG symptoms and their effects on daily activities, with scores ranging from 0 to 24, where higher scores indicate more impact of MG on daily activities, which is quick to complete (<10 min), and can be used in clinical trials or routine clinical practice [11]; a revised 15-item myasthenia gravis quality of life scale (MG-QOL15r)—an MG-specific quality of life measure containing 15 patient-reported items, with resulting scores ranging from 0 to 30, where higher scores indicate a worse health-related quality of life [12]; and the myasthenia gravis composite scale (MGC)—a hybrid of patient- and physician-reported test items that measure symptoms and signs of MG, where for each item, there are four weighted response options [13]. Optional instruments in clinical practice with MG patients are: MGFA post-intervention status (MGFA-PIS)—measures post-intervention status but is not sensitive to small changes in MG status, and does not define improvement or worsening and instead relies on QMG or MGC criteria, representing the evaluator’s comprehensive clinical judgment, independent of any predefined threshold for change in outcome measure scores [14,15]; MGFA therapy status—a descriptive tool of current treatment regimens in MG patients [14]; and MG manual muscle testing (MG-MMT)—represents the sum of strength scores across muscle groups, with each group graded from 0 (normal function) to 4 (complete paralysis) [14].

### 3.2. Current Treatment

Treatment options in MG are symptomatic medications (acetylcholinesterase inhibitors, e.g., pirydostigmine) and immunosuppressive therapy like glucocorticosteroids, azathioprine, ciclosporin A, methotrexate, mycophenolate mofetil, tacrolimus or cyclophosphamide [16,17,18,19]. In MG crisis, which is the most dangerous condition, intravenous immunoglobulin (IVIG) and plasmapheresis (PLEX) are used and are similarly effective [20,21]. Another treatment strategy to achieve remission is biological treatment (e.g., rituximab, ravulizumab, eculizumab, zilucoplan, efgartigimod alfa, rozanolixizumab, nipocalimab, and inebilizumab). Rituximab is a monoclonal antibody against the CD20 protein that leads to B-cell depletion and to the synthesis of new antibody-secreting plasma cells [22]. In a randomized clinical trial of a single infusion with rituximab (RTX) vs placebo, the proportion of patients exhibiting minimal disease manifestations, maintained on low-dose corticosteroids and without requiring rescue therapy at 4 months, was 71% in the rituximab group compared with 29% in the placebo group, reflecting a statistically significant difference [23]. Also, in long-term follow-ups of this clinical trial, disease activity and treatment burden, including hospitalizations and rescue therapies, remained low, suggesting a potential long-term benefit of rituximab on disease progression [24]. Ravulizumab is a humanized monoclonal antibody, which binds to human complement protein C5, inhibiting its division into C5a and C5b, thus preventing the cascade of complement proteins that leads to the destruction of the postsynaptic neuromuscular junction membrane by the membrane attack complex (MAC) [25,26]. The mechanism of eculizumab is familiar to ravulizumab—binding with high affinity to human terminal complement protein C5 [27]. Zilucoplan, like ravulizumab and eculizumab, is a complement C5 inhibitor, but is a macrocyclic peptide that can be self-administered as a once-daily subcutaneous injection [28]. Efgartigimod alfa is a human IgG1 antibody Fc-fragment, which binds to the neonatal Fc receptor (FcRn). The FcRn receptor is a molecule that recycles IgG, extending its half-time by about four times that of other immunoglobulins that are not recycled by FcRn; IgGs bound to FcRn are saved from lysosomal degradation and released at physiological pH outside the cell [29]. That is why the function of the FcRn receptor boosts the concentration and amount of immunoglobulins, which is pathognomonic in autoimmune diseases like MG. When the efgartigimod links with the FcRn receptor, it competitively reduces endogenous IgG, thereby reducing IgG recycling and increasing IgG degradation [30]. Rozanolixizumab target is also a region of FcRn, reversibly inhibiting IgG salvage and recycling, accelerating IgG catabolism by the lysosomal degradation pathway, thus reducing IgG concentrations in patients with acetylcholine receptor (AChR) or muscle-specific kinase (MuSK) autoantibody-positive generalized myasthenia gravis [31]. In 2025 another FcRn inhibitor was approved for the treatment of generalized MG. Nipocalimab is a fully human monoclonal antibody that binds to and inhibits the neonatal Fc receptor (FcRn), leading to a reduction in circulating IgG levels for the treatment of generalized MG in patients aged ≥12 years who are positive for anti-AChR or anti-MuSK antibodies [32]. Inebilizumab, previously known in the treatment of anti-AQP4+ neuromyelitis optica spectrum disorders (NMOSDs), is an anti-CD19 monoclonal antibody, which depletes CD19+ B-cells, which are central to disease pathogenesis, and was approved at the end of 2025 by the FDA to treat adult patients with anti-AChR+ or anti-MuSK+ generalized MG [10,33].

With all these emerging options, several evolving treatment guidelines were proposed that differ between the countries. The international consensus guidelines introduced in 2016 aimed to help to promote the greater harmonization of MG treatment. These guidelines statements were formulated for the management of symptomatic and immunosuppressive therapies, intravenous immunoglobulin and plasma exchange, impending and manifest myasthenic crisis, thymectomy, juvenile myasthenia gravis, MG associated with antibodies against muscle-specific tyrosine kinase, and myasthenia gravis in pregnancy [34]. The 2020 update of those guidelines updated previous recommendations for thymectomy; new recommendations were developed for the use of rituximab, eculizumab, and methotrexate, as well as for early immunosuppression in ocular MG and in MG associated with immune checkpoint inhibitor therapy [16]. However, MG still represents a highly unpredictable condition that urgently needs a novel biomarker discovery that would aid in the optimization of its management protocols.

## 4. Extracellular Vesicles

### 4.1. Origin, Function and Biomarker Potential

Extracellular vesicles (EVs) are membrane-bound particles released by cells. One of the major EV subtypes are exosomes, typically defined as small vesicles of approximately 40–200 nm in diameter that are actively secreted into the extracellular milieu. EVs carry diverse bioactive cargo, including proteins, lipids, mRNA, miRNA, lncRNA, and DNA, thereby contributing to the regulation of cellular homeostasis and mediating intercellular and interorgan communication. It should be noted, however, that EVs represent a heterogeneous population of vesicles, and in the current literature the term “exosomes” is often used without strict adherence to standardized isolation and characterization criteria. Therefore, in this review, we use the term “EVs” as a general descriptor where appropriate, while retaining the original terminology used in the cited studies. In addition, variability in EV isolation and characterization methods may contribute to the differences observed across studies.

Exosomes are ubiquitously present in body fluids and exert their biological effects through autocrine, paracrine, and endocrine signaling pathways [35]. They are involved in the intercellular transportation of materials [36] and provide a mechanism for long distance intercellular communication. It has been demonstrated that cells transfected with a plasmid encoding enhanced green fluorescent protein (eGFP) release exosomes containing eGFP RNA. When these cells were injected into mice, the RNA was detected not only in the circulation but also in various other cell types, indicating direct interaction between exosomes and recipient cells [37]. Due to their small size and lipophilic nature, exosomes are taken up by a number of organ tissues and cell populations. The role of exosomes is proven in the pathophysiology of different autoimmune diseases and cancers. In multiple sclerosis (MS) significant data have been generated demonstrating the role of exosomes in modulating immune cell transmigration across the blood–brain barrier (BBB). In response to pro-inflammatory cytokines such as TNF, IFN-γ, and IL-1, endothelial cells—as well as leukocytes, microglia, astrocytes, and platelets—were shown to release exosomes containing metalloproteinases and caspase-1. These enzymes are known to promote blood–brain barrier (BBB) disruption and facilitate the transmigration of lymphocytes and myeloid cells into the central nervous system (CNS) [38]. Studies on exosomes in multiple sclerosis (MS) patients have shown a general increase in their levels in both serum and cerebrospinal fluid (CSF) in individuals with relapsing–remitting MS, particularly during relapse, compared to patients with progressive forms of the disease and healthy controls [39]. Furthermore, in experimental studies of MS exosomes isolated outside CNS tissue, expressed myelin proteins and the presence of myelin oligodendrocyte glycoprotein (MOG) correlated strongly with disease activity. Exosomes may contribute to the amplification and persistence of anti-myelin immune responses in multiple sclerosis (MS) and hold potential as novel biomarkers of disease activity [40].

Exosomes, which carry molecular information from their parental cells and are present in multiple body fluids, represent highly promising biomarkers for liquid biopsy [41]. The exosome-based liquid biopsy is non-invasive, because it requires the collection, e.g., of blood, urine or other body fluid samples, without a tissue biopsy. Furthermore, it allows for dynamic monitoring with multiple sampling over time, enabling the tracking of the disease course and treatment response. Also, exosomes are a wealth of biomarkers, containing molecular signatures specific to output cells, which increases diagnostic sensitivity. Thanks to this, analyzing their content enables the non-invasive monitoring of pathological changes, detecting cancer at an early stage or tracking the response to therapy. The liquid biopsy of exosomes has the biggest potential in oncology, cardiovascular diseases and neurology. For example, a multicenter, population-based, retrospective, case–control study for establishing exosome-based diagnostic signature to facilitate blood-based, early detection of patients with gastric cancer have shown that 10-miRNA signature (Destinex) successfully identified early-stage (pT1) gastric cancer. Furthermore, there was a significant decrease in miRNA expression levels in postsurgery serum samples, confirming the high specificity of the panel [42].

### 4.2. Role of Exosomes and Their Cargo

Current isolation methods do not allow for the precise separation of EV subtypes. Common approaches, including differential ultracentrifugation and size exclusion chromatography, together with emerging techniques such as microfluidic platforms and affinity-based methods, are often time-consuming and may yield EV preparations of variable purity, with a limited ability to fully resolve vesicle heterogeneity. Consequently, no single method for the separation and characterization of EV subpopulations meets all ideal criteria [43]. Despite these limitations, numerous studies suggest that exosomes function as nano-sized transport vesicles that facilitate intercellular communication through the transfer of proteins, lipids, nucleic acids, and metabolites to recipient cells. Notably, the molecular cargo of exosomes appears to depend on the cellular origin and the biological context of communication.

The most common proteins found in exosomes belong to classes involved in membrane transport and fusion. Other frequently identified proteins include cytoskeletal, metabolic, signaling, and carrier proteins, as well as albumin. Among exosomal proteins, tetraspanins are particularly prominent—including CD63, CD9, CD81, and CD82. Additional commonly detected proteins include ESCRT-I–associated proteins (e.g., Tsg101), lysosome-associated membrane glycoproteins (LAMP-1 and LAMP-2B), multivesicular body-associated protein Alix-1, heat shock proteins (Hsp60, Hsp70, and Hsp90), adhesion molecules (CD45 and CD11b), major histocompatibility complex (MHC) class I and II molecules, Rab GTPases, and membrane-binding proteins such as annexins. Also, exosomes carry a variety of enzymes, including GTPases and metabolic enzymes such as peroxidases, pyruvate kinases, lipid kinases, and enolase-1 [44].

Exosomes play an important role in cell–cell communication by carrying DNA, microRNAs, mRNAs, and circRNAs [45]. A microarray analysis of exosomal RNA reveals that exosomes released by human mast cells contain approximately 1300 distinct RNA species. These exosomes transport RNA, including microRNAs (miRNAs), into recipient cells—some of which have been demonstrated to retain functional activity [46]. Among small RNA species, microRNAs (miRNAs) are relatively more enriched in exosomes compared to their parent cells. miRNA expression was examined in multiple cell lines and their derived exosomes, demonstrating that specific miRNAs (e.g., miR-150, miR-142-3p, and miR-451) are preferentially sorted into exosomes [47].

Taking into account the analysis of lipids, exosomes are especially rich in glycosphingolipids, sphingomyelin (SM), cholesterol, and phosphatidylserine (PS), which together constitute a significant molar percentage of their total lipid content [48]. The lipidomic analysis of the metastatic prostate cancer cell line PC-3 and its secreted exosomes quantified approximately 280 lipid species across 18 distinct lipid classes [49].

Exosomes have long been postulated as a potential source of novel biomarkers. They may serve as minimally invasive biomarkers for diagnosis, disease activity monitoring, or treatment response in many medical conditions. In sepsis exosomes content including proteins (e.g., lactylated/acetylated HMGB1; eCIRP; mtDAMPs; Galectin 9; SPTLC3; IL-6; IL-10; Tetraspanin markers; TREM2; APN; NHE3; uATF3; GBP2; NAD(P)H oxidase; and AQP-4) and miRNAs represent a valuable source of biomarker candidates due to their stability, widespread occurrence, and ability to reflect disease states. Their potential in detecting sepsis-induced organ damage—such as cardiomyopathy, acute kidney injury, and acute lung injury—is particularly noteworthy, as they offer real-time insights into cardiac and renal dysfunction [50]. In oncology exosomes derived from cancer cells are enriched in differentially expressed proteins and nucleic acids, and have been explored as novel biomarkers for the early detection, staging, and prognosis of various cancers (e.g., ovarian cancer, breast cancer, gastric cancer, HCC, PDAC, colorectal cancer, NSCLC, and prostate cancer), underscoring their significant potential in liquid biopsy and precision oncology [51]. Scientific reports indicate that exosomes cargo can be useful in the screening of neurodegenerative diseases. Neuronal and astrocyte-derived exosomes have been recognized as optimal candidates for biomarker screening in Alzheimer’s disease (AD). A diagnostic model for Alzheimer’s disease (AD) was developed using data from an initial pilot study (*n* = 40; 20 controls, 20 AD patients), followed by validation in a second, larger dataset (*n* = 114; 56 controls, 58 AD patients). A receiver operating characteristic (ROC) curve analysis identified a panel of six proteins capable of distinguishing AD patients from healthy controls with high accuracy. This panel included five upregulated proteins—Ig-like domain-containing protein (A0A0G2JRQ6), complement C1q subcomponent subunit C (C1QC), complement component C9 (CO9), platelet glycoprotein Ib beta chain (GP1BB), and Ras suppressor protein 1 (RSU1) and one downregulated protein, disintegrin and metalloproteinase domain-containing protein 10 (ADA10). Furthermore, linear correlation analysis demonstrated a significant association between this protein combination and cognitive performance. That is why the plasma exosomal protein panel comprising A0A0G2JRQ6, C1QC, CO9, GP1BB, RSU1, and ADA10 represents a novel candidate biomarker for distinguishing Alzheimer’s disease (AD) patients from healthy individuals [52].

## 5. Role of Exosomes in MG

### 5.1. Pathogenesis

It is believed that exosome cargo (exosome proteins and non-coding RNA including micro RNAs) can contribute to the initiation of autoimmunity in myasthenia gravis (MG). Exosomes can transfer autoantigens (e.g., fragments of the acetylcholine receptor—AChR) and present them to immune cells, thus enhancing the activation of the T and B lymphocytes responsible for the production of autoantibodies. Exosomes derived from thymic epithelial cells and dendritic cells may disrupt immune tolerance and promote the survival of autoreactive T lymphocytes. Exosomes contain miRNAs that influence lymphocyte differentiation (e.g., Th17 and Treg), which may shift the balance toward a pro-inflammatory response and promote the maintenance of a chronic autoimmune process. There is no strong evidence that exosomes directly damage the neuromuscular junction, but they may exacerbate local inflammation and indirectly increase autoantibody-mediated damage to the AChR, MuSK, or LRP4 receptors. They contribute to MG pathogenesis by acting as messengers that transfer immune-modulating molecules like microRNAs (miRNAs) and proteins, which can promote inflammation and autoantibody production. They are involved in central tolerance disruption in the thymus by presenting antigens. Exosomes can carry antigen-presenting molecules and tissue-specific antigens from the thymus, potentially disrupting central tolerance. Exosomal miRNAs and lncRNAs may also play a critical role in the pathophysiology of MG [53]. In thymoma-associated myasthenia gravis (TAMG) expression of miR-125a-5p was found to be negatively correlated with Foxp3 expression in normal tissues adjacent to thymomas in TAMG patients. Additionally, these findings demonstrated that miR-125a-5p regulates Foxp3 gene expression in Jurkat cells. Collectively, these results indicate that dysregulated miR-125a-5p expression and its modulatory effect on Foxp3 may contribute to the pathogenesis of TAMG [54].

**Figure 1:**
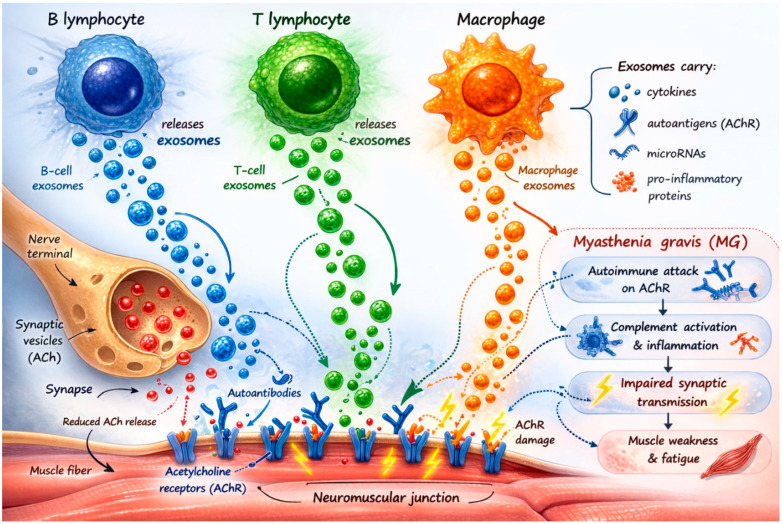
MG pathogenesis and the role of exosomes. MG originates from the action of pathogenic autoantibodies directed against a muscle’s receptors, mainly AChR. Circulating exosomes released from various immune cells involved in the autoimmune reaction leading to MG may provide clues on the status of these processes in the course of the condition. Solid arrows indicate direct interactions, whereas dashed arrows represent indirect or regulatory interactions. This figure was generated using an AI tool. The authors take full responsibility for its content.

The dysregulation of circulating micro-RNAs (miRNAs), such as miR-29a/b1, miR-7, let-7a-5p, let-7f-5p, and miR-27a-3p family miRNA, and the aberrant methylation of tumor suppressor genes have also been associated with the initiation and progression of MG and TAMG, respectively [55]. The putative role of exosomes in MG pathogenesis has been summarized in Figure 1.

### 5.2. Diagnostic and Biomarker Potential of Exosomes in MG

In myasthenia gravis (MG), specific patterns of increased circulating miRNAs have been identified and proposed as potential disease biomarkers. In acetylcholine receptor antibody positive (AChR+) MG patients, elevated miRNAs are miR-150-5p, miR-21-5p, and miR-30e-5p. In this study, an analysis of the regulatory mechanisms of these miRNAs by integrating chromatin immunoprecipitation sequencing (ChIP-seq) data from the Encyclopedia of DNA Elements (ENCODE), aiming to elucidate the transcription factor pathways governing their expression in myasthenia gravis (MG), was made from 73 MG patients [56]. Interestingly, inflammatory activation facilitated the exosomal packaging of the AChR+ specific miRNAs miR-21-5p and miR-30e-5p, which may explain the upregulated circulating levels of these miRNAs observed in MG patient serum [56]. Furthermore, it has been found that levels of miR-30e-5p were higher in patients with ocular AChR+ myasthenia gravis who later progressed to generalized MG, compared to those who remained with only ocular symptoms. That is why the expression of miR-30e-5p can have the prediction potential of clinical disease course [57]. In the study of MG AChR+ patient sera, hsa-miR-150-5p—known to promote T cell differentiation—and hsa-miR-21-5p—a key regulator of Th1/Th2 immune responses—were significantly upregulated. In contrast, hsa-miR-27a-3p, which plays a role in natural killer (NK) cell cytotoxicity, was found to be downregulated [58]. In muscle-specific tyrosine kinase antibody positive (MuSK+) MG patients, upregulated miRNA are miR-151a-3p, miR-423-5p, let-7a-5p, and let-7f-5p [56]. Chinese scientists published that in the study of plasma samples from 92 patients with MG, exosomal microRNA miR-106a-5p levels were significantly decreased in ocular and generalized MG patients compared with healthy controls and were associated with patient quantitative MG scores (QMGSs). The area under the curve (AUC) values for hsa-miR-106a-5p were 0.728 and 0.813 in ocular and generalized MG patients, respectively. Moreover, the exosomal expression of miR-106a-5p was found to be much more decreased in the moderate–severe MG group—1.2 ± 0.4 (QMGS ≥ 8 or MG with respiratory failure)—compared with that in the mild MG group—2.7 ± 0.4 (QMGS < 8) (*p* < 0.001) [59]. miR-106a-5p dysregulation has been reported in the T cells of patients with immune diseases (e.g., MS), and may inhibit the proliferation, migration, and invasion of carcinoma cells (melanoma, colorectal and gastric cancer and esophageal squamous-cell carcinoma) by modulating various mRNA targets [59,60]. The microRNA miR-150-5p is known as a biomarker in MG due to its increase in the serum of patients and its decrease after thymectomy, correlated with an improvement of symptoms. The study evaluating the effect of thymectomy on disease-specific microRNA (miRNA) biomarkers in 80 patients with MG enrolled in the prospective randomized MGTX trial demonstrated that, compared with baseline (6.2 ± 1.3), circulating miR-150-5p levels were significantly reduced 24 months after thymectomy (5.2 ± 1.2; *p* = 0.0011). This reduction was accompanied by a significant improvement in clinical status, as reflected by the decrease in quantitative myasthenia gravis (QMG) scores (*p* < 0.001) [61]. Several studies suggest that the presence of ectopic thymic tissue in patients with myasthenia gravis (with a reported median prevalence of approximately 41%) is a significant predictor of poorer outcomes following thymectomy. Patients in whom ectopic thymic tissue persists after thymectomy appear to have less favorable clinical outcomes compared with those without residual ectopic thymic tissue [62]. Monitoring circulating miRNAs may potentially provide indirect information on residual immunological activity related to persistent or ectopic thymic tissue in selected patients. In this context, miRNA levels could be explored as adjunctive biomarkers reflecting the biological effect of thymectomy, although this application remains hypothetical and requires further validation. An upregulation of miR-150-5p expression in 40 early-onset AChR-positive MG patients’ thymuses was observed, which correlated with the presence of thymic B-cells. In situ hybridization experiments further revealed that miR-150-5p was predominantly expressed by cells located in the mantle zone of germinal centers (GCs) of the thymuses. However, any correlation between the degree of thymic hyperplasia and the serum levels of miR-150-5p in MG patients was not observed. Also, the expression of miR-150-5p was found to be downregulated, particularly in CD4+ T cells, when compared to healthy controls. This suggests that the elevated serum levels of miR-150-5p may originate from its release by activated peripheral CD4+ T cells [63]. Increased expression of miR-150 in MG thymuses was associated with a decreased expression of *MYB*, the most well-known miR-150 mRNA target, which is highly expressed in the thymus [63,64]. In the thymus of patients with MG, miR-150 can be secreted by B-cells and alter *MYB* expression locally and consequently affect T cells [60,65]. MYB is characterized by an altered expression in autoimmune diseases, because of the early regulation of T cells [63,66]. In the study to determine predictive biomarkers associated with the risk of conversion from ocular myasthenia gravis (OMG) to generalized myasthenia gravis (GMG), in 13 patients who progressed to SGMG apart from miR-30e-5p, the level of miR-150-5p was significantly higher in patients with secondary GMG (SGMG) compared to OMG patients with late onset of MG (7.4 ± 1.1 vs. 6.4 ± 1.1; *p* = 0.01) [57].

miR-125a-5p has been reported to play an important role in cancer and immune diseases. In the thymic tissues from thirteen TAMG patients, the levels of miR-125a-5p were remarkably increased with a 6.72-fold change relative to the normal thymus controls [54].

A study investigating the extraction and sequencing of miRNAs from the serum exosomes of patients with early-onset ocular myasthenia gravis (OMG), generalized myasthenia gravis (GMG), and healthy controls revealed that several serum exosomal miRNAs—particularly miR-4712-3p, miR-320d, and miR-3614-3p—are differentially expressed among these groups. These miRNAs are involved in key biological processes such as dendritic development and cell adhesion, and are also closely linked to axon guidance and the mTOR signaling pathway. Consequently, miR-4712-3p, miR-320d, and miR-3614-3p may serve as valuable diagnostic biomarkers for patients with early-onset OMG [67].

Exosomes in seronegative myasthenia gravis (MG) represent a promising avenue for biomarker discovery, particularly in patients who lack classical autoantibodies, including anti-acetylcholine receptor (AChR), anti-MuSK, and anti-LRP4 antibodies. A study of small extracellular vesicle (sEV)-derived miRNAs (sEV-miRNAs) from 17 patients with pediatric MG (10 AChR-Ab seropositive MG; 7 AChR-Ab seronegative MG) showed that twenty-four sEV-miRNA were differentially expressed in pediatric myasthenia gravis (MG) patients compared to healthy controls. Among autoantibody seronegative myasthenia gravis patients, the significantly downregulated miR-143-3p was validated using quantitative real-time polymerase chain reaction analysis. This analysis provides a potential biomarker in the diagnostic evaluation of pediatric patients with seronegative myasthenia gravis [68].

Detecting the lncRNA profiles of serum exosomes in six MG patients (the subjects were selected based on established diagnostic criteria for myasthenia gravis, including characteristic clinical manifestations, fatigable muscle weakness with a positive neostigmine test, electrophysiological evidence of impaired neuromuscular transmission on single-fiber electromyography, and AChR antibody status—positive or negative) and six healthy controls found a total of 378 lncRNAs to be significantly upregulated and 348 significantly downregulated in MG patients compared to healthy controls. Among these, the top five lncRNAs (NR_104677.1, ENST00000583253.1, NR_046098.1, NR_022008.1, and ENST00000581362.1) were validated and confirmed to be markedly elevated in the serum exosomes of MG patients. Notably, the expression level of NR_046098.1 was positively correlated with MG severity [69].

Collectively, these findings suggest that exosome-derived non-coding RNAs may provide complementary diagnostic information in seronegative MG; however, current evidence remains limited by small cohort sizes and requires validation in larger, independent studies.

**Figure 2:**
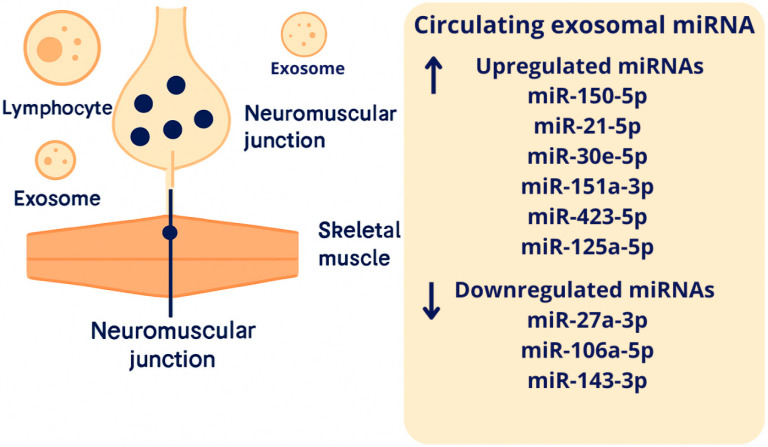
Circulating exosomal miRNAs and their diagnostic potential in MG. The concentration of specific miRNA sequences may be elevated or decreased in myasthenia gravis and serve as a diagnostic marker of this disease. Upward arrow (↑) indicate upregulated levels, whereas downward arrow (↓) indicate downregulated levels of specific exosomal miRNA sequences in myasthenia gravis. The figure was generated using an AI-based tool and further modified and formatted using Canva. The authors take full responsibility for the final content.

The diagnostic potential of miRNAs selection in myasthenia gravis is presented in Figure 2.

A Chinese prospective, open-label, and self-controlled pilot trial of single, low-dose (600 mg) infusion of rituximab (RTX) in 12 patients with acetylcholine receptor antibody positive (AChR+) refractory myasthenia gravis (MG) showed that RTX reduced levels of the serum exosomal miR-150-5p by 47.9% (−0.70 ± 0.89, *p* = 0.006), in addition to miR-146a-5p (−2.03 ± 8.57, *p* = 0.570). The efficiency of RTX was reflected in three out four clinical scale scores, which were significantly decreased after six months: MG activities of daily living (MG-ADL) by 43.9% (*p* = 0.022); MG-specific manual muscle testing (MMT) by 67.4% (*p* = 0.019); and MGFA-quantitative myasthenia gravis (MGFA-QMG) by 27.8% (*p* = 0.019). The last clinical scale, MG-specific quality of life (QOL-15), did not show a statistically significant reduction, but a downward trend was observed. Patients’ requirement of oral GCS (prednisolone) was also reduced by 43% (*p* = 0.018) [70].

An additional clinically relevant question is whether patients with ocular MG (OMG) and elevated miR-150-5p might benefit from early B-cell-depleting therapy in order to prevent generalization of the disease. Given the association of miR-150-5p with B-cell-related immune activity in MG, this hypothesis is biologically plausible. However, at present there is not enough direct clinical evidence supporting the use of miR-150-5p as a biomarker for guiding early B-cell-targeted interventions, such as rituximab, in OMG patients. In particular, prospective studies evaluating whether biomarker-driven early B-cell depletion can modify the risk of progression from ocular to generalized MG are lacking. Therefore, this remains an open research question that warrants investigation in future prospective, stratified clinical studies.

Furthermore, in another Chinese study to investigate the efficacy of low-dose (600 mg over 2 consecutive days, 100 mg on day 1 and 500 mg on day 2) rituximab (RTX) in 12 MG patients with antibodies against muscle-specific tyrosine kinase (MuSK+), a single dose of RTX after six months reduced serum exosomal miR-151a-3p by 28.1% (*p* = 0.031), while there was no statistically significant difference in let-7a-5p, let-7f-5p, or miR-423-5p expression levels. RTX lowered scoring in five clinical severity scores: MG-MMT from 13.50 ± 10.50 to 1.25 ± 1.49 (*p* = 0.010); QMG from 11.83 ± 5.29 to 2.33 ± 3.63 (*p* = 0.010); QOL-15 from 25.17 ± 14.40 to 7.50 ± 9.92 (*p* = 0.010); ADL from 7.83 ± 4.43 to 1.17 ± 1.85 (*p* = 0.010); and MGC from 12.58 ± 7.45 to 2.33 ± 3.50 (*p* = 0.010). Six out of twelve patients reached asymptomatic status (QMGS = 0). RTX reduced the daily dosage of prednisone from 27.29 ± 20.71 mg to 12.29 ± 11.45 mg; the daily dosage was reduced to <10 mg in 6/12 patients (*p* = 0.032) [71]. Also, there are scientific reports that a high expression of miR-151a-3p is described as a poor prognosis in patients’ plasma with gastric cancer with liver metastasis (GC-LM) [72].

Exosome-derived biomarkers hold considerable promise for guiding treatment selection and monitoring therapeutic responses to targeted therapies, including B-cell depletion, complement inhibition, and neonatal Fc receptor (FcRn) blockade. As components of liquid biopsy approaches, they may provide minimally invasive, real-time molecular insights into disease activity and treatment-related immunological changes, thereby enabling more precise therapeutic adjustments compared with conventional non-specific immunosuppressive strategies. The molecular cargo of exosomes, including proteins, miRNAs, and other RNAs, can reflect the mechanistic effects of targeted interventions, such as alterations in complement pathway components following complement inhibition or immune cell-associated signatures after B-cell depletion. Furthermore, dynamic changes in exosomal content may allow the early detection of molecular relapse or treatment resistance, including shifts in immune-regulatory markers or autoantibody-associated signatures, potentially informing optimal timing for re-dosing or therapeutic switching. Their suitability for repeated sampling makes them particularly attractive for the longitudinal monitoring of chronic autoimmune diseases treated with biologics, including complement inhibitors and FcRn blockers.

Identified miRNAs and lncRNAs and their diagnostic and biomarker potential in myasthenia gravis (MG) are summarized in Table 1.

### 5.3. Therapeutic Perspectives of Exosomes in MG

In experimental studies of autoimmune myasthenia gravis (EAMG) in mice, exosomes produced from microRNA-146a overexpressing dendritic cells (DCs) expressed decreased levels of molecules CD80 and CD86, altered T helper cell profiles from Th1/Th17 to Th2/Treg both in serum and spleen, and suppressed clinical course of MG in mice. In dendritic cells (DCs) transfected with microRNA-146a, the expression levels of CD80, CD86, and MHC molecules were 62.62 ± 16%, 49.35 ± 16.3%, and 55.34 ± 13.9%, respectively (*p* < 0.05), compared to 85.29 ± 13.89%, 80.96 ± 14.62%, and 85.57 ± 13.42% in the control DCs. Additionally, the secretion levels of pro-inflammatory cytokines were significantly decreased in DCs transfected with microRNA-146a compared to control cells. The levels of IL-6 and IL-12p70 in the supernatants of dendritic cells (DCs) transfected with microRNA-146a were 73.00 ± 12.33 and 317.43 ± 28.42, respectively, compared to 129.16 ± 26.03 and 462.5 ± 58.39 in control DCs (both *p* < 0.05). Furthermore, the therapeutic effects of those exosomes were partly dose-dependent and antigen-specific [73].

Another exploratory study showed that exosomes derived from atorvastatin-modified bone marrow dendritic cells (BMDCs) (statin-Dex) in experimental autoimmune myasthenia gravis (EAMG) in mice can suppress the clinical symptoms of EAMG rats. There is more medical evidence that statins have an inhibiting impact on the expression and secretion of pro-inflammatory cytokines, T cell activation, proliferation and the maturation and function of APCs. A flow cytometric analysis showed that statin-Dex had a lower level of MHC class II and higher level of FasL when compared with control-Dex (*p* < 0.001). Moreover, statin-Dex demonstrated immunomodulatory activity in vivo by downregulating the expression of CD80, CD86, and MHC class II molecules on endogenous dendritic cells. The rats in the statin-Dex group presented lower clinical scores when compared with the rats in the control-Dex group (*p* < 0.05 and *p* < 0.01) and the control group (*p* < 0.05). These results showed that Dex with a lower level of MHC class II and higher levels of FasL and IDO protein could contribute to the protective effects in EAMG. These effects were connected with upregulated levels of indoleamine 2,3-dioxygenase (IDO)/Treg cells and partly dependent on the FasL/Fas pathway, which finally resulted in the reduced synthesis of IgG2a, IgG2b and anti-R97-116 IgG antibodies [74].

Exosomes from statin-modified bone marrow dendritic cells (statin-Dex) promote the expansion of thymus-derived natural regulatory T cells in experimental autoimmune myasthenia gravis (EAMG). The effects of exosomes on co-stimulatory surface markers and the autoimmune regulator (Aire), which is a transcriptional regulator expressed in distinct thymic stromal cell populations, including cortical and medullary thymic epithelial cells (cTECs and mTECs) as well as thymic dendritic cells (tDCs), were evaluated by Chinese scientists in EAMG rats. Statin-Dex upregulated Aire expression in the thymus in vivo. Moreover, in the presence of thymic stromal cells in vitro, statin-Dex promoted the differentiation of Foxp3^+^ natural regulatory T (nTreg) cells among thymocytes. Collectively, these findings suggest that the increased Aire expression induced by statin-Dex may contribute to enhanced Foxp3^+^ nTreg cell development within the thymus [75].

Mouse bone marrow (BM)-derived immature dendritic cell-derived exosomes (iMDEXs) induce tolerance in a mouse model of experimental autoimmune myasthenia gravis (EAMG). Treatment with iMDEX_(1000)_ markedly reduced disease severity in EAMG, as indicated by significantly decreased clinical scores relative to the PBS control group across all time points (*p* < 0.05). iMDEX_(1000)_ ameliorated the progression of EAMG by reducing AChR-reactive lymphocyte proliferation (*p* < 0.05), AChR antibody levels (AChR-reactive IgG1, IgG2a, IgG2b and IgG3) (*p* < 0.05) and pro-inflammatory cytokine levels (IFN-γ, TNF-α and IL-6) (*p* < 0.05) [76]. Therapeutic perspectives of exosomes in MG are condensed in Table 2.

### 5.4. Limitations and Future Directions of Exosomes in MG

Despite increasing evidence supporting the role of exosomes in myasthenia gravis, several limitations should be acknowledged. Current studies are largely based on small and heterogeneous patient cohorts, with limited stratification of MG subtypes and clinical phenotypes. In addition, methodological variability in exosome isolation and characterization contributes to inconsistencies across studies, limiting the direct comparability of findings. Most available data are cross-sectional in design, with relatively few longitudinal studies evaluating dynamic changes in exosome cargo in relation to disease progression or treatment response. Furthermore, a substantial proportion of evidence is derived from preclinical models, which may not fully recapitulate the complexity of human disease. Future studies should focus on large, multicenter, longitudinal cohorts with standardized protocols for exosome isolation and characterization. The integration of complementary analyses of exosome cargo, including RNA- and protein-based profiling, may improve biomarker robustness and mechanistic understanding of disease activity. Importantly, prospective clinical studies are needed to validate exosome-derived biomarkers for diagnosis, disease stratification, and treatment monitoring in well-defined MG patient populations.

## 6. Conclusions

Exosomes in myasthenia gravis (MG) are increasingly investigated in the context of immune regulation and disease-associated processes. They may serve as a minimally invasive source of biomarkers with potential utility for diagnosis and disease characterization. In addition, exosomes may provide insights into mechanisms underlying MG and could support future research on disease monitoring and immune modulation. However, their precise functional roles in MG remain to be fully elucidated. Although the field of exosomes in MG is rapidly evolving, further clinical, experimental, and translational studies are required to clarify their relevance in disease pathophysiology and to evaluate their potential applications.

## Figures and Tables

**Table 1:** Identified miRNAs and lncRNAs as potential biomarkers in myasthenia gravis (MG). Upward arrows (↑) indicate increased expression, whereas downward arrows (↓) indicate decreased expression of identified miRNAs and lncRNAs.

miRNA/lncRNA	MG Subtype	Sample Matrix	Cohort Size	Direction of Expression Change	Main Biological Functions/Pathways	Clinical Significance	References
miR-150-5p	AChR+ MG	Serum, thymus tissue, CD4+ T cells, serum exosomes	73 MG patients; 80 MGTX trial; 12 RTX-treated AChR+ MG	↑ in serum & thymus; ↓ in CD4+ T cells; ↓ after RTX	T cell differentiation; MYB regulation	Biomarker; correlates with disease activity; decreases after thymectomy and RTX	[56,58,61,70]
miR-21-5p	AChR+ MG	Serum, serum exosomes	73 MG patients	↑	Th1/Th2 regulation; inflammation; exosomal transport	Potential biomarker	[56,58]
miR-30e-5p	AChR+ MG; OMG→GMG conversion	Serum, serum exosomes	73 MG patients; 13 OMG→GMG	↑	Inflammatory response; exosomal packaging	Predicts conversion from OMG to GMG	[56,57]
miR-27a-3p	AChR+ MG	Serum	Not reported	↓	NK cell cytotoxicity	Indicates impaired innate immunity	[58]
miR-151a-3p	MuSK+ MG	Serum exosomes	12 RTX-treated MuSK+ MG	↑ (baseline); ↓ after RTX	Muscle gene regulation	Biomarker; decreases after RTX	[56,71]
miR-423-5p	MuSK+ MG	Serum exosomes	12 RTX-treated MuSK+ MG	↑	Metabolism, proliferation	Potential biomarker	[56,71]
let-7a-5p, let-7f-5p	MuSK+ MG	Serum exosomes	12 RTX-treated MuSK+ MG	↑	Inflammation, gene regulation	Potential biomarkers	[56,71]
miR-106a-5p	AChR+ OMG & GMG	Plasma exosomes	92 MG patients	↓	Cell proliferation, migration	Correlates with severity (QMGS); AUC 0.728–0.813	[59]
miR-125a-5p	TAMG	Thymus tissue	13 TAMG patients	↑ (6.72×)	Cancer & immune regulation	Highly expressed in TAMG thymus	[54]
lncRNAs (NR_104677.1, ENST00000583253.1, NR_046098.1, NR_022008.1, ENST00000581362.1)	Seronegative & seropositive MG	Serum exosomes	6 MG patients + 6 controls	↑	Immune regulation	NR_046098.1 correlates with severity	[69]
miR-4712-3p, miR-320d, miR-3614-3p	Early-onset OMG/GMG	Serum exosomes	Not reported	Differentially expressed	Dendritic development, adhesion, axon guidance, mTOR	Diagnostic biomarkers	[67]
miR-143-3p	Pediatric MG (AChR+ and seronegative)	sEV-miRNA	17 pediatric MG patients	↓	sEV-miRNA dysregulation	Biomarker for pediatric seronegative MG	[68]

**Table 2:** Summary of therapeutic perspectives of exosomes in myasthenia gravis. Upward arrows (↑) indicate increased expression, whereas downward arrows (↓) indicate decreased expression of specific molecules, and rightward arrow (→) indicate transition or phenotypic shift between cell types or states.

Study	Type of Exosomes/Modification	Model	Effects on DCs/Immunological Markers	Cytokines/Th Profile	Clinical Effects	Mechanism/Notes
[73]	miR-146a-overexpressing DCs	Mouse EAMG	↓ CD80 (62.62 ± 16%), ↓ CD86 (49.35 ± 16.3%), ↓ MHC (55.34 ± 13.9%) vs. control	Shift Th1/Th17 → Th2/Treg	Suppression of MG progression, partly dose- and antigen-specific	Reduction in pro-inflammatory cytokines, immunomodulation
[74]	Statin-Dex (atorvastatin + BMDCs)	Rat EAMG	↓ CD80, ↓ CD86, ↓ MHC II, ↑ FasL	-	Lower clinical scores vs control and control-Dex	Effects linked to ↑ IDO/Treg and partly FasL/Fas, ↓ IgG2a, IgG2b, anti-R97-116 IgG
[75]	Statin-Dex	Rat EAMG	↑ Aire in thymus, effects on cTEC, mTEC, tDC	Promoted Foxp3^+^ nTreg development in thymus	-	Supports natural Treg development via Aire
[76]	iMDEX (immature BMDC-derived exosomes)	Mouse EAMG	-	↓ AChR-reactive lymphocyte proliferation	↓ EAMG clinical scores, reduced disease progression	↓ AChR IgG1/IgG2a/IgG2b/IgG3, ↓ IFN-γ, TNF-α, IL-6

## Data Availability

No new data were created or analyzed in this study. Data sharing is not applicable to this article.

## Abbreviations

The following abbreviations are used in this manuscript:

AChR	Acetylcholine receptor
BMDC	Bone marrow-derived dendritic cells
CNS	Central nervous system
DC	Dendritic cells
EAMG	Experimental autoimmune myasthenia gravis
EV	Extracellular vesicles
FcRn	Neonatal Fc receptor
GMG	Generalized myasthenia gravis
IDO	Indoleamine 2,3-dioxygenase
IFN-γ	Interferon gamma
IgG	Immunoglobulin G
IL	Interleukin
IVIG	Intravenous immunoglobulin
lncRNA	Long non-coding RNA
LRP4	Low-density lipoprotein receptor-related protein 4
MG	Myasthenia gravis
MG-ADL	Myasthenia gravis activities of daily living scale
MGFA	Myasthenia Gravis Foundation of America
MGC	Myasthenia gravis composite scale
MG-QOL15r	Revised 15-item myasthenia gravis quality of life scale
miRNA	MicroRNA
MuSK	Muscle-specific kinase
PLEX	Plasma exchange
QMG	Quantitative myasthenia gravis score
RTX	Rituximab
TAMG	Thymoma-associated myasthenia gravis
Treg	Regulatory T cells
